# Addition of Tolcapone in Intrajejunal Levodopa Infusion Therapy Requires a Pronounced Dose Reduction

**DOI:** 10.1002/mdc3.13029

**Published:** 2020-08-18

**Authors:** Nils Schröter, Sabine Ahrendt, Anna Hager, Michel Rijntjes

**Affiliations:** ^1^ Clinic of Neurology and Neurophysiology, Medical Center–University of Freiburg, Faculty of Medicine, University of Freiburg Freiburg Germany

**Keywords:** LCIG, COMT‐inhibition, tolcapone, Parkinson's disease

Continuous jejunal application of levodopa–carbidopa intestinal gel (LCIG) in patients with advanced Parkinson's disease leads to a decrease in both *off* time and dyskinesia^1^; however, 60% of these patients still require concomitant oral dopaminergic medication consisting of levodopa and dopamine agonists.^2^

When oral dopaminergic therapy is supplemented by tolcapone, a catechol‐*O*‐methyl‐transferase (COMT) inhibitor, a dose reduction of 30% is recommended by the manufacturer. On the other hand, a systemic review suggested reducing the oral dose by as much as 50%.^3^

In a single pilot study, tolcapone and another catechol‐*O*‐methyl‐transferase inhibitor, entacapone, were administered in addition to LCIG, with a target dose reduction of 20%; however, based on pharmacological considerations, the authors recommended a 40% dose reduction for cotreatment with tolcapone.^4^ Apart from this preliminary data, the extent to which LCIG can or should be reduced after the addition of tolcapone to LCIG has not been further investigated in a clinical setting.

We now describe 4 consecutive patients with Parkinson's disease with motor fluctuations who received off‐label LCIG therapy 24 hours a day as a result of severe, painful hypokinesia at night; a nighttime dose reduction was not possible because of persistent hypokinesia (Table 1). One patient still in need of extra boluses had a total LCIG intake of 96.4 mL per day, whereas 3 cases had daily LCIG doses ranging from 101.8 to 116.0 mL requiring more than 1 cassette per day. To reduce LCIG consumption and hypokinetic periods, all 4 patients (who each had normal liver function) received supplementary tolcapone (100 mg) 3 times per day. Tolcapone was chosen instead of entacapone or opicapone because of its expected stronger effect on the daily LCIG dose—especially with regard to the expected increase in LCIG demand during the course of the disease. Despite an initial LCIG dose reduction of 10% to 40%, all patients suffered from both hyper‐ and dyskinesia, whereas 2 older patients additionally experienced delirium with changes in circadian rhythm, increased agitation and confusion, delayed discharge from hospital, and the need for pharmacotherapy with quetiapine that was discontinued in 1 patient after discharge. All negative side effects ceased after LCIG dosage was gradually reduced by 19% to 50% during a period of 3 to 10 days. One patient only required a dose reduction of 19%, possibly attributed to the disease running an especially long and mild course and also because this particular patient was the only 1 among the 4 with a tremor‐dominant subtype. Following LCIG dose reduction, the severity of motor symptoms did not differ between baseline LCIG dosage and the last dose of dopaminergic therapy, as documented by the Movement Disorder Society–Unified Parkinson's Disease Rating Scale Part III. All patients continued to take tolcapone in 2‐ to 9‐month follow‐ups, and in 1 case a dose adjustment of LCIG was necessary. Our report shows that tolcapone can be used to reduce LCIG intake to 1 cassette in patients who would otherwise require more than 100 mL daily. However, based on our experience, a dose reduction of up to 50% should be aimed for which is considerably more than previously suggested to prevent negative side effects. For determining the optimal dosage and treating potential side effects, we recommend applying supplementary tolcapone therapy in an in‐patient setting only. An early reduction in LCIG infusion rate should be considered to mitigate the risk of delirium, especially in older patients.

**TABLE 1:** *Patient characteristics*

		Disease Duration, y						MDS‐UPDRS III	Follow‐Up, mo
Case	Age, y	Duration of LCIG Therapy, mo	LCIG Dose Before Addition of Tolcapone	LCIG Dose at Time of Discharge	Relative Dose Reduction, Total %	Time Required Until Final Dose Reduction	Side Effects Prior to Final Dose Reduction	H&Y Subtype	LCIG Dose MDS‐UPDRS III
1	82	12	CIR: 3.6 mL/h	CIR: 2.0 mL/h		3 d	Severe hyperkinesia	Admission: 63 Discharge: 60	* phone 9
		13	MD: 7 mL ED: 3 mL	MD: 3 mL ED: 1 mL	**46**		Hyperactive delirium	H&Y: 3	52 mL No change in clinical condition
			**Total: 96.4 mL**	**Total: 52 mL**				Akinetic‐rigid	
2	59	11	CIR: 4.5 mL/h	CIR: 2.2 mL/h		3 d	Severe hyperkinesia	Admission: 51 Discharge: 48	3 57.8 mL
		0	MD: 5 mL ED: 3 mL	MD: 5 mL ED: 0 mL	**50**		On dystonia	H&Y: 3	45
			**Total: 116 mL**	**Total: 57.8 mL**				Akinetic‐rigid	
3	74	19	CIR: 3.7 mL/h	CIR: 3.1 mL/h		3 d	Hyperkinesia	Admission: 27 Discharge: 28	7
		0	MD: 10 mL ED: 3 mL	MD 8 mL ED: 0 mL	**19**		On dystonia	H&Y: 3	82.4 mL
			**Total: 101.8 mL**	**Total: 82.4 mL**				Tremor dominant	23
4	88	11	CIR: 3.7 mL/h	CIR: 2.1 mL/h		10 d	Severe hyperkinesia	Admission: 43 Discharge: 38	2
		0	MD: 8 mL ED: 6 mL	MD: 3 mL ED: 0 mL	**48**		Hyperactive	H&Y: 4	67 mL
			**Total: 102.8 mL**	**Total: 53.4 mL**			Delirium	Akinetic‐rigid	32

LCIG, levodopa–carbidopa intestinal gel; MDS‐UPDRS III, Movement Disorder Society ‐ sponsored revision of the Unified Parkinson’s Disease Rating Scale Part III; H&Y, Hoehn & Yahr; CIR, continuous infusion rate; MD, morning dose; ED, extra dose.

Bold data, Cumulative daily Intake of LCIG and relative dose reduction of daily LCIG intake

Our observations are potentially useful in view of increasing pressure to reduce costs, the predicted rise in the number of patients with Parkinson's disease,^5^ and, most important, for practical purposes in patients with a high LCIG demand.

## Author Roles

(1) Research Project: A. Conception, B. Organization, C. Execution; (2) Manuscript Preparation: A. Writing of the First Draft, B. Review and Critique.

N.S.: 1A, 1B, 1C, 2A

S.A.: 1B, 2B

A.H.: 1B, 2B

M.R.: 1A, 1B, 2B

## Disclosures

**Ethical Compliance Statement**: The local ethics committee of the University of Freiburg stated that no formal approval was needed due to the format of this clinical vignette. Common ethical guidelines were followed. Written informed consent was obtained and documented by all patients. We confirm that we have read the Journal's position on issues involved in ethical publication and affirm that this work is consistent with those guidelines.

**Funding Sources and Conflicts of Interest**: No specific funding was received for this work. The authors declare that there are no conflicts of interest relevant to this work.

**Financial Disclosures for the Previous 12 Months**: Nils Schröter received funding from Berta‐Ottenstein‐Programme for Clinician Scientists, Faculty of Medicine, University of Freiburg. Sabine Ahrendt, Anna Hager, and Michel Rijntjes declare that there are no additional disclosures to report.
